# Dopamine Agonist Prescribing in a Gambling Disorder Patient Population: A Clinical Audit to Determine Prevalence in the NHS Northern Gambling Service

**DOI:** 10.1192/bjo.2025.10338

**Published:** 2025-06-20

**Authors:** John Barker, Rebecca Lees

**Affiliations:** NHS Northern Gambling Service, Leeds, United Kingdom

## Abstract

**Aims:** Dopamine agonists are prescribed to treat several major physical and mental illnesses. Rotigotine, ropinerole, pramipexole (full agonists) are routinely used to treat symptoms of restless leg syndrome, whilst aripiprazole (partial agonist) is used as both an antipsychotic and mood stabiliser.

There is significant co-morbidity between gambling disorder and those psychiatric presentations resulting in the prescribing of aripiprazole. Full and partial dopamine agonists are known to increase the risk of de-novo gambling disorder, and exacerbation of existing gambling disorder. The aims of the audit were thus as follows:

To ensure that 100% of initial assessments include a full medication history, comprising current dopamine agonist (full or partial) prescribing history and indication for prescribing.

To ensure that 100% of patients identified at referral as prescribed dopamine agonists are screened by a psychiatrist within the service prior to allocation to the appropriate gambling disorder treatment pathway.

**Methods:** 402 initial assessments were carried out in the service in 2023. A random sample of 30 was selected using a random number generator and the initial assessments extracted from the Trust electronic record system (Care Director). Initial assessments were screened by a CT3 doctor to ascertain if a complete drug history was documented. The presence of dopamine agonist prescribing was noted including the drug name. Where dopamine agonists were prescribed, medical records were referenced to check if a screen was conducted by a psychiatrist.

**Results:** 53% (16/30) of the sample had a full drug history documented. 17% (5/30) of the sample were prescribed a dopamine agonist and in all cases, this was aripiprazole. Of the 5 patients in the sample prescribed aripiprazole, only 1 was documented as being referred for screening by a psychiatrist prior to commencement of psychological treatment for gambling disorder.

**Conclusion:** 53% of records sampled had a full drug history documented, suggesting that there are further patients who were prescribed dopamine agonists at the point of initial assessment that the service was unaware of. Of the 17% of the sample that were prescribed dopamine agonists, all cases were aripiprazole. This highlights the significant psychiatric co-morbidity and the importance of screening by a psychiatrist to exclude the dopamine agonist as a causative factor in the presentation.

Further staff training on the clinical importance of dopamine agonists in this context, ensuring 100% of initial assessments include a complete drug history, and discussion with a psychiatrist where appropriate are recommended to improve patient care within the service.

